# Case of a Polypus Uteri

**Published:** 1800-01

**Authors:** Thomas Denman

**Affiliations:** Old Burlington Street


### Medical and Phyfical Journal.

VOL. III.] JANUARY, 1800. [NO. XI.

### *Cafe of a Polypus Uteri, communicated by* Dr. Denman.*

IN the beginning of July laft, I was defired to vifit a foreign lady, who was born, and had paffed the greateft part of her life, in a hot climate. She was between thirty and forty years of age, had been early married, but never had a child. Her ftrength was much reduced, her afpect was wan, and her lips and gums pale, but her appetite and digeftion were good. She informed me, that about three years ago, fhe had fuffered much from a profufe uterine hæmorrhage, and from that, to the time of my converfing with her, had never paffed a fingle day without a return of the hæmorrhage in a greater or lefs degree, and fometimes it had been very confiderable. She had never fuffered any uterine pain, or other local inconvenience, except a frequent inclination to void her urine, and fome difficulty in voiding it. When fhe was free from the hæmorrhage, fhe had a watery or ichorous difcharge of an unpleafant kind. She had been under the care of feveral medical gentlemen, who had not given a decided opinion on her cafe, but had generally made an alarming prognoftic. She had received little or no benefit from a variety of medicines, which had been prefcribed for her, and which fhe had taken with great perfeverance.

She permitted me to take an examination, and I was very much furprized to find the whole *vagina* and *uterus*, as far as I could reach, (for the *os uteri* was completely dilated) filled with a flefhy fubftance of a pretty firm texture, which could not be fufpected to be any thing but a *polypus*, though it was not poffible to difcover the ftem or pedicle of it.

As the ftate of this patient was fuch as to excite great apprehenfion for her fafety, from the continuance of the hæmor-

* We take the liberty of pointing out to our junior correfpondents, Dr. Denman's manner of relating cafes as worthy of imitation.—Ed.

hage, and as there was no likelihood of this being ſtopped, without the extirpation of the *polypus*, the neceſſity of removing it was evident. Yet, from its magnitude, there muſt clearly be much difficulty in paſſing a ligature over it; and if this difficulty were ſurmounted, it would be doubtful on what part it might be fixed, as it was not certain that the *uterus* was not inverted. But from the failure to paſs the ligature, though I might be diſappointed, no harm could ariſe; and if it were paſſed, and happened to fix upon a part unable to bear its operation, I could deſiſt immediately on the appearance of any untoward ſymptom; it was therefore thought right to make the attempt. I was foiled in the firſt trials I made; but on July the ninth, I ſucceeded in paſſing the ligature, and conducted it by the help of a long wooden probe, with a perforation at the end, far beyond the reach of my fingers; ſo as to include the whole circumference of the tumour. On the firſt day I only drew the ends of the ligature ſufficiently tight, to keep it fixed upon the part to which it was conducted; but I drew it tighter on every ſucceeding day till the ſeventeenth, when the ligature came away. From the time of my fixing the ligature, there had been a profuſe ſanious diſcharge, plainly ſhewing that the tumour was decaying; yet, though it was detached, no attempts were made to extract it; but it was ſuffered to remain, with the expectation of its bulk being leſſened by putrefaction. On the nineteenth, after ſome diſturbance in the bowels, ſhe complained of ſevere pains in the region of the *uterus*, returning at ſhort intervals like the pains of labour, raiſing the ſame kind of effort as is made to expel a child. During the continuance of a pain, I found the *polypus* preſſing firmly upon, and dilating the *os externum*, which was rigid and much contracted. In about four hours, with the little aſſiſtance I was able to give, the *polypus* was excluded without any hæmorrhage, either during the time of its expulſion or afterwards.

Dr. Baillie, who ſaw this patient with me, had the *polypus*, which, notwithſtanding its diminution, (it was much decayed) weighed two pounds and three ounces; ſo that it does not ſeem unreaſonable to ſuppoſe it muſt originally have weighed three pounds, being far the largeſt I ever removed.

The lady recovered without one unpleaſant ſymptom, menſtruated naturally at the end of five weeks, and has ſince remained in perfect health.

*Old Burlington Street,*

*Nov.* 27, 1799.

THOMAS DENMAN.

